# The Role of Wnt Signal in Glioblastoma Development and Progression: A Possible New Pharmacological Target for the Therapy of This Tumor

**DOI:** 10.3390/genes9020105

**Published:** 2018-02-17

**Authors:** Mariachiara Zuccarini, Patricia Giuliani, Sihana Ziberi, Marzia Carluccio, Patrizia Di Iorio, Francesco Caciagli, Renata Ciccarelli

**Affiliations:** 1 Department of Medical, Oral and Biotechnological Sciences, University of Chieti-Pescara, via dei Vestini 29, 66100 Chieti, Italy; mariachiara.zuccarini@unich.it (M.Z.); patricia.giuliani@unich.it (P.G.); sihana.ziberi@unich.it (S.Z.); marzia.carluccio@unich.it (M.C.); patrizia.diiorio@unich.it (P.D.I.); f.caciagli@unich.it (F.C.); 2 Aging Research Center and Translational Medicine (CeSI-MeT), via L. Polacchi 11, 66100 Chieti, Italy; 3 StemTeCh Group, via L. Polacchi 11, 66100 Chieti, Italy

**Keywords:** glioblastoma multiforme, epithelial-to-mesenchymal transition, Wnt signal pathway, drugs targeting Wnt pathway

## Abstract

Wnt is a complex signaling pathway involved in the regulation of crucial biological functions such as development, proliferation, differentiation and migration of cells, mainly stem cells, which are virtually present in all embryonic and adult tissues. Conversely, dysregulation of Wnt signal is implicated in development/progression/invasiveness of different kinds of tumors, wherein a certain number of multipotent cells, namely “cancer stem cells”, are characterized by high self-renewal and aggressiveness. Hence, the pharmacological modulation of Wnt pathway could be of particular interest, especially in tumors for which the current standard therapy results to be unsuccessful. This might be the case of glioblastoma multiforme (GBM), one of the most lethal, aggressive and recurrent brain cancers, probably due to the presence of highly malignant GBM stem cells (GSCs) as well as to a dysregulation of Wnt system. By examining the most recent literature, here we point out several factors in the Wnt pathway that are altered in human GBM and derived GSCs, as well as new molecular strategies or experimental drugs able to modulate/inhibit aberrant Wnt signal. Altogether, these aspects serve to emphasize the existence of alternative pharmacological targets that may be useful to develop novel therapies for GBM.

## 1. Introduction: General Outline of the Wnt Pathways

The Wnt pathway is currently recognized as an important regulatory signal able to influence developmental embryonic processes [1] and to modulate self-renewal, maintenance and differentiation of adult tissue stem cells [2,3]. About three decades ago, it was discovered that the proto-oncogene *Int-1* caused the malignant transformation of mouse mammary tissue, once activated by insertion of the mouse mammary tumor virus (MMTV). Moreover, *Int-1* resulted to be a homolog of *Drosophila wingless* (*wg*), which in turn controls segment polarity during larval development [4]. Thus, the gene was named *Wnt1* (wingless-type MMTV integration site family member 1).

The Wnt pathway is present in the entire animal kingdom. In mammals, at least 19 glycolipoproteins and a great number of receptors have been discovered so far. The interaction between these different proteins and their own receptors leads to a great variety of responses in the cell [5]. The Wnt system is usually divided into canonical (β-catenin-dependent) and non-canonical (β-catenin-independent) Wnt pathways. Briefly, in the canonical pathway, β-catenin, which is a central player of this signaling cascade, is entrapped into a protein complex formed by Axin, glycogen synthase kinase-3 (GSK-3), casein kinase 1 (CK1) and adenomatous polyposis coli (APC). This complex favors β-catenin degradation by proteasomal ubiquitination. When a Wnt protein binds to receptors of the frizzled (FZD) and low-density lipoprotein receptor related protein (LRP5/6) families, the degradation complex results to be inhibited [6,7], even though the levels of functionally active β-catenin may be increased by additional Wnt signal-independent mechanisms [8]. Thus, β-catenin accumulates, enters the nucleus and activates genes acting as a co-activator of the transcription factors belonging to the T cell factor/lymphoid enhancer factor-1 (TCF/LEF1) family [9].

The non-canonical Wnt signaling cascade comprises several different pathways depending on the major intracellular mediators involved, such as a dishevelled (DVL)-c-Jun N-terminal kinase (JNK) pathway and several Wnt/Ca^2+^ pathways identified so far [10].

The DVL-JNK pathway is also called planar cell polarity (PCP) pathway, as it is involved in cellular polarity and cytoskeletal modulation of *Xenopus* embryos [11]. Binding of Wnt proteins to the FZD receptors activates, via DVL, small GTPases, Rho and Rac, and JNK kinase. The activation of this signaling cascade leads to changes in the cytoskeleton and activation of transcription factors of activator protein-1 (AP-1) family.

In the non-canonical Ca^2+^-mediated pathways, Wnt proteins bind to FZD receptors activating DVL and phospholipase C (PLC) [12,13]. The resulting inositol 1,4,5-triphosphate (IP3) interacts with Ca^2+^ channels of the endoplasmic reticulum (ER) membrane stimulating intracellular Ca^2+^ ion release, which in turn activates a great number of kinases/factors such as protein kinase C (PKC),- cell division cycle 42 (Cdc42) [14], Ca^2+^/calmodulin-dependent protein kinase II (CaMKII), transforming growth factor (TGFβ) activated kinase (TAK1), nemo-like kinase (NLK) [15,16] and/or calcineurin (CaCN)-nuclear factor of activated T cells (NFAT) [17].

Traditionally, FZD-LRP receptor-co-receptor combinations have been considered canonical, whereas the non-canonical Wnt pathway is highly complex, given that Wnt ligands may interact not only with FZD but also with other receptors [18], such as tyrosine kinase-like orphan receptor 1/2 (ROR1/2), receptor-like tyrosine kinase (RYK), protein tyrosine kinase 7 (PTK7) and van gogh-like 1/2 (VANGL1/2) [19]. Since there are more than 15 different Wnt receptors and co-receptors, and their expression may vary from cell to cell, the interaction between one of these receptors and a given ligand results in a great variety of effects. Indeed, the activity of many Wnt proteins is influenced by the investigated cell type and the receptors expressed in those cells. Consequently, it is highly complicated to foresee with certainty and/or to interpret the response to a stimulus induced by Wnt cascade activation.

Nonetheless, a recent study has established a critical role for this pathway in brain development and function so that a dysregulation of Wnt system may lead to the onset of tumors, including glioblastoma multifome (GBM) [20]. The latter is the most lethal cancer of human adult brain since the current therapy is inadequate [21]. The therapeutic failure in treating this tumor has probably brought to the publication of an impressive number of papers in the last five years, concerning the study of new druggable targets involved in GBM growth and progression, among others the Wnt pathway. Since the recurrence of GBM is likely due to the presence of a population of cancer stem cells (CSCs) named GBM stem cells (GSCs), with features similar to normal neural stem cells (NSCs), here we examined the role of Wnt signaling in the processes involved in the growth and maturation, up to the adult life, of the nervous system formation, or in the development and expansion of GBM. For this reason, we tried to highlight the role of several factors associated to Wnt system dysregulation in this tumor, thereby supporting GBM onset and progression. Finally, we discussed the possibility to consider those factors as alternative pharmacological targets for the therapeutic management and control of this tumor.

## 2. Wnt Signal in Brain Development and Adult Function

Wnt signal plays a key role during embryonic development of different tissues and organs, including the nervous system [22]. It is known that the formation of specific structures in the neural tube is due to a sequential process wherein the nervous tissue, deriving from ectoderma, is induced to acquire typical characteristics of cerebral anterior regions, whereas posterior characteristics are subsequently promoted in the anterior neural tissues by posteriorizing factors. Interestingly, the activation of the Wnt pathway needs to be tightly regulated during neural developmental processes to allow a correct formation and regionalization of different brain areas [23]. In this context, several lines of evidence indicate that Wnt signals act to caudalize the developing neural tube [24], while Dickkopf-1 (DKK1), an inhibitor of Wnt signaling, induces anterior brain structures [25,26,27]. In other words, the posteriorizing function of Wnts must be inhibited to allow generation of anterior neural structures [28]. Evidence in this sense has been accumulated in different animal models, including *Drosophila* larvae [29], chick embryos [30], zebrafish [31,32], rodents [33,34] and humans [28].

During brain development, Wnt system, together with other signaling molecules (NOTCH, fibroblast growth factor (FGF)) directs the commitment of neural cell precursors from the subventricular zones (SVZ) [35]. The activity of Wnt/β-catenin pathway has been associated with proliferation of neural progenitor cells in early phases of brain development [36], whereas it inhibits self-renewal capacity of the same cells and promotes their neuronal differentiation in later phases [37,38,39]. Wnts indeed regulate diverse neural events including dendrite formation and synaptic function. This complex process comprises neuronal migration and polarization, axon guidance and dendrite development [40]. Indeed, in *Drosophila*, Wnt signals are important for modulating, together with FGF and JNK, the axonal extension and retraction to achieve precise neuronal connectivity and generation of the wiring pattern [41]. Furthermore, several experiments have been carried out in the model of the zebrafish brain, in which it was demonstrated that genes for Wnt7, an important regulator of brain synaptogenesis, is highly expressed in a number of central nervous system (CNS) CNS structures at different developmental stages of patterning and neural specification [42]. Interestingly, the non-canonical Wnt pathway, including Wnt5 and the transmembrane PCP protein Vangl2, is mostly involved in the control of cell movements, which are governed by polarized cell behaviors [43]. Recent studies performed also in chicks and frogs confirmed that the non-canonical Wnt–PCP pathway plays a major role in neural crest migration. Planar cell polarity signaling controls contact inhibition of locomotion between neural crest cells by localizing different PCP proteins at the site of cell contact during collision and locally regulating the activity of Rho GTPases [44]. Through these signals, the Wnt system contributes to the development of different cerebral structures in mammalians, in particular cortex and hippocampus [37,39].

Finally, recent findings pointed out a crucial activity of the Wnt pathways in regulating also the function of mature neurons as well as the integrity of the brain. Indeed, in addition to the expected roles in adult neurogenesis, wherein a major role of Wnt signaling has been reported in proliferative regions such as the SVZ [45], Wnt signaling results to be essential for neuronal survival and for the regulation of higher brain function in adults, i.e., by supporting pre- and postsynaptic function and transcriptional regulation [46,47,48]. Wnt pathways appear to have particular importance in specific brain regions. For example, the canonical pathway upregulates the expression of the Ca^2+^ channel Cav3.1 in the thalamus, leading to enhanced T-type Ca^2+^ currents [49]. Moreover, some data indicate a critical requirement of Wnt signaling for the normal function of midbrain dopaminergic neurons reviewed by [50,51] as well as of hippocampal excitatory glutamatergic and inhibitory GABAergic neurotransmission [52,53].

By considering the findings reported above on the involvement of Wnt system in supporting the adult brain function and integrity, it is expectable that deregulation of this signal cascade contributes to the pathogenesis of neurological and psychiatric diseases. Indeed, behavioral and cognitive defects have been reported in adult mice with genetically modified Wnt signaling components [54,55]. In humans, Wnt pathway activity plays important roles in the hippocampal neurogenesis and is progressively lost during ageing [54]. Moreover, a remarkable relationship between an impaired Wnt signal and neuronal damage has been reported in Alzheimer’s Disease [56,57]. Perturbed Wnt signals are also involved in other neurodegenerative diseases including altered myelination [58] and loss of dopaminergic neuron function associated with hereditary forms of Parkinson’s Disease [59,60] or schizophrenia [61].

## 3. Cancer Stem Cells in Neuro-Oncology

Mounting evidence suggest that tumor growth and recurrence are mainly due to the presence of a small population of cells inside or surrounding the cancer tissue, displaying characteristics very similar to normal stem cells, so that they are called CSCs. Although the debate is still under way, the CSC model is receiving confirmation from numerous studies on peripheral and central nervous system tumors [62,63,64]. Cancer stem cells are characterized by a great ability of self-renewal, a feature shared with normal stem cells. However, this property may be exaggerated in CSCs resulting indefinitely prolonged. This condition favors the accumulation of mutations, giving place to initiation of the tumor and, subsequently, supporting its growth and disease progression [64]. In the case of brain cancers, CSCs seem to derive from NSCs. Some factors, which are present only in cells during brain development, may cause NSC transformation into CSCs. This could be the case of *LIN28A*, a microRNA-regulating protein, the expression of which is high during the early stages of neural tube development and decreases over time [65]. An increased expression of LIN28A has been reported in some aggressive pediatric brain tumors [66,67] and also in GBM and related cells lines [68]. Noteworthy, LIN28A transfection in human NSCs facilitates their tumor transformation mediated by Kirsten Ras (KRas), a pro-oncogenic signal [68].

Neural stem cells have been isolated from different regions of the adult human brain, even though they are largely concentrated in the SVZ, from which they can move, in case of injury, to replace damaged cells and/or to eliminate migratory tumor cells. In this way, they contribute to maintain homeostasis in the adult human brain [69]. This migratory capacity, coupled to the preference for white matter and capillary basement membranes, is also observed in glioma cells, indicating that NSCs and tumor cells share a similar behavior and motility. Recently, Sancho-Martinez et al. [70], by inducing a genetic manipulation of p53 and receptor tyrosine kinase signaling in NSCs obtained by human-induced pluripotent stem cells, demonstrated the acquisition of CSC-like features in vitro, including enhanced self-renewal and migratory properties, as well as the possibility to generate human glioma-like upon orthotopic transplantation of 500 cells into the murine brain. Thereby, it is now widely accepted that these cells are involved in the origin and recurrence of GBM [64]. The association of GBM with the SVZ may remarkably influence patients’ survival [71].

However, the involvement of NSCs in tumors is not limited to gliomas. Indeed, they have the potential to give rise to other brain tumors such as medulloblastoma, which prevailingly arises from granule neuron precursors present in the cerebellum or also from stem cells in the dorsal brainstem [72]. Similarly, neural progenitors from different regions of the central nervous system form various subtypes of ependymomas with different properties [73].

**Figure 1:**
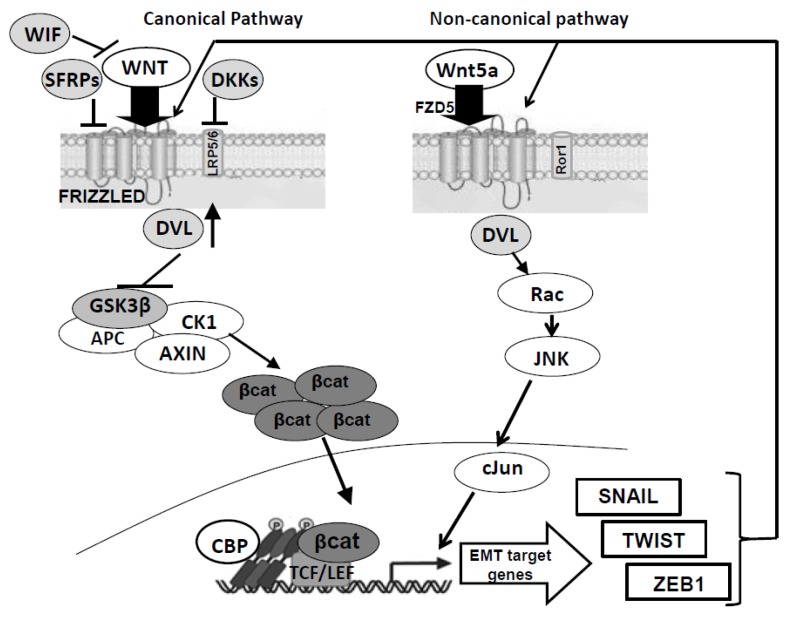
Relationship between epithelial-to-mesenchymal transition (EMT) and the Wnt pathway in glioblastoma-derived stem cells (GSCs). As discussed in the text of the review, a possible relationship seems to exist between the EMT process, which favors the self-renewal, migration and resistance to therapy of cancer stem cells, including GSCs, and the Wnt signals, which, when deranged, as it occurs in GSCs, can activate/cooperate with EMT downstream factors (Snail, Twist, and Zeb1) creating a vicious cycle able to potentiate the activity of upstream factors. The explanation for the abbreviations used are the following: histone acetyltransferase CREB binding protein (CBP); WIF: Wnt inhibitory factor; SFRP: Secreted frizzled-related protein; DKK: Dickkopf; DVL: Dishevelled; GSK3β: Glycogen synthase kinase 3 β; CK: Casein kinase; APC: Adenomatous polyposis coli; TCF/LEF: T-cell factor/lymphoid enhancing factor; JNK: Jun N terminal kinase.

Cancer stem cells not only support the growth of tumors but are also implicated in their invasiveness as they may undergo a process called epithelial-to-mesenchymal transition (EMT), when it occurs in epithelial cancers. Epithelial-to-mesenchymal transition causes biochemical changes inducing a mesenchymal phenotype in epithelial-derived CSCs, thus enhancing their migration and resistance to apoptosis [74,75,76]. In CSCs deriving from GBMs, which are classified as neuroepithelial tumors [77], a similar process can be observed leading to the so-called glial-mesenchymal transition (GMT) [77,78]. Similar to EMT, GMT is related to cell migration and tumor spread by evoking single-cell movement [79]. This process has also been associated with the resistance of GBM to the current chemo- and radio-therapy [80]. Interestingly, recent studies demonstrated the existence of a relationship between EMT and the Wnt pathway in CSCs, even though it is not fully understood [81]. As for GBM, Kahlert et al. [82] showed that the modulation of Wnt signaling altered the expression of EMT activators and, more recently, another group showed that resveratrol, a drug able to impair GSC proliferation and motility by decreasing the expression of some Wnt signaling pathway-related genes (in particular, c-Myc and β-catenin), also reduced the protein levels of Twist1 and Snail1, two pivotal activators of EMT program [83] (see Figure 1).

## 4. Alteration of Wnt Pathways in Glioblastoma Multiforme and Derived Stem Cells

While Wnts are deeply involved in maintaining the stemness of normal stem cells, thus contributing to control tissue repair/regeneration, emerging data indicate that dysregulation of this pathway supports the onset of CSCs, which, as reported above, assure the enlargement of the tumoral mass and eventually the spread of metastases [84].

Focusing on GBM and related GSCs, it emerges that alterations of the Wnt pathway may be considered as a discriminating factor between normal and malignant cells in the adult human brain. Indeed, several studies revealed that the expression and nuclear localization of β-catenin and its transcription factor TCF4 are significantly higher in glioma compared to normal brain tissue, and these findings positively correlate to World Health Organization (WHO) glioma grade [85]. By examining data from the Cancer Genome Atlas [86], which classified GBMs into four distinct molecular subtypes named Proneural, Neural, Classical and Mesenchymal, the heavy influence of dysregulated Wnt signal emerges for some of them, in particular for the proneural and the mesenchymal subgroups. In the former subgroup, characterized by a high tumor incidence in younger patients and a bad prognosis, it has been reported an elevated expression of two Wnt pathway activators, TCF4 and SOX, [87,88]. Moreover, in the latter subgroup, high levels of canonical Wnt factors such as DKK1, FZD1 and LEF1 were found to be associated with very poor clinical outcome [89]. Oncogenic activities, such as proliferation, inhibition of apoptosis and invasion, have also been coupled to abnormal Wnt/β-catenin signaling in glioma cell lines and a few studies, performed on primary GSC cultures, confirmed these data [89,90,91]. Altogether, these findings indicate that the WNT system plays a fundamental role in gliomagenesis affecting a large variety of cellular processes.

However, reports regarding ligands, receptors and mechanisms responsible for altered Wnt signaling in GBM and GSCs are so numerous that a systematic classification of the results is highly complicated. By trying to accomplish this task, we found that, for example, it is still unclear the involvement of one or both (canonical and non-canonical) Wnt pathways in GBM onset/progression. Concerning the canonical Wnt signal, it has been reported that:

- It is responsible for GBM resistance to chemo- and radio-therapy [80,92].
- It supports GBM invasion [82]. In particular LEF1, a downstream factor in the canonical Wnt pathway, plays a key role in stem cell maintenance and EMT process, promoting cell migration and invasion of different cancer types including GBM [93]. In the same way, HOXA13, belonging to the Homeobox (HOX) gene family, promotes glioma progression in part via Wnt- and TGF-β-induced EMT and, similar to LEF1, is a potential diagnostic biomarker for GBM and an independent prognostic factor in high-grade glioma [94].
- It is related to worse prognosis [95].
- It is referred to as a characteristic feature of GSCs, by contributing to maintain stem cell properties [90,91].

On the other hand, the non-canonical Wnt signaling is more closely related to the invasiveness of GBM that the canonical one. Indeed, several components of the PCP arm of non-canonical Wnt pathway including VANGL1, VANGL2 and FZD7 are transcriptionally upregulated in glioma and correlate with poor patient outcome. Consequently, while knocking down VANGL1 suppresses the motility of GBM cell lines, restoration of NRDP1, a RING finger type E3 ubiquitin ligase whose decrease in GBM correlates with poor prognosis, reduces GBM cell migration and invasiveness by suppressing PCP signaling. These findings pointed out an important mechanistic role for this pathway in GBM malignancy [96]. In addition, RYK, an atypical member of the receptor tyrosine kinase (RTK) family involved in the control of neuronal differentiation [97], resulted to be essential for WNT5a-dependent invasiveness in glioma, and its expression correlated with the WHO histological classification for glioma tissues [98]. Additionally, it has recently been reported that RYK contributes in establishing the stem-like phenotype of GBM cells by regulating β-catenin expression and function [99].

Another aspect to consider is that while genetic mutations in Wnt pathway factors seem to be the prevalent cause of aberrant function of this molecular cascade in peripheral tumors (i.e., colorectal cancer and hepatocellular carcinoma) and also in medulloblastoma, this is not the rule in GMB [100]. Except for a homozygous mutation of FAT apical cadherin 1 (FAT1), an inhibitor factor of Wnt signal present in about 20% of GBM, epigenetic alterations would represent the most important causes of Wnt dysfunction in this tumor.

These alterations regard several factors, most of which, when normally expressed, are able to inhibit the Wnt cascade [101]. Some of them are strictly related to the Wnt system. This is the case of the Wnt inhibitory factor 1 (WIF-1), an inhibitor of the canonical Wnt pathway since it hinders binding of Wnt proteins to the FZD/LRP receptor. Its expression is down-regulated in nearly 40% of all GBMs [102]. Moreover, it has recently been shown that WIF1 selectively down-regulates the Wnt/Ca^2+^ pathway and this effect was coupled to a significant reduction of p38-mitogen-activated protein kinase (p38-MAPK) phosphorylation. In agreement with the regulatory function of the Wnt/Ca^2+^ pathway on migration and invasion, WIF1 expression inhibited cell migration in vitro and in an orthotopic brain tumor model. In contrast, loss of WIF1 enhanced the activity of the metastasis-associated lung adenocarcinoma transcript 1 (MALAT1), a long non-coding RNA and a key positive regulator of tumor invasion [103].

Similarly, DKK1 and secreted frizzled-related proteins (SFRPs), other canonical Wnt pathway antagonists, seem to be epigenetically inactivated in GBM. In particular, DKK1 is expressed at low levels in a glioma cell line U87-MG and in GSCs deriving from three different patients in comparison with bone marrow mesenchymal stem cells [104]. The human achaete-scute homolog (ASCL1), another critical factor in the Wnt pathway, repressed DKK1, thus revealing to be essential for the tumorigenicity of GSCs in vivo [105].

Several members of SFRPs, a family of soluble proteins, negatively modulate the Wnt signaling cascade, and it has been demonstrated that their aberrant expression is present in different types of cancer. In GBM, SFRP4 was 15 times up-regulated and SFRP1 was 360 times down-regulated in GSCs from 9 different tumors and this event was associated with a very remarkable reduction in median patient survival. Therefore, it is not surprising that treatment of primary GSC cultures with recombinant SFRP1 halted cell cycling and induced apoptosis [106]. Furthermore, the exposure of GBM cell lines to SFRP4 sensitized these cells to the pharmacological treatment with temozolomide (TMZ), a classic anti-GBM drug, improving the efficacy of this chemotherapeutic agent [107]. In addition, SFRP3 raised a certain interest since results obtained by immunohistochemistry, digital scanning and image analysis showed statistically significant differences between its expression levels and glioma malignancy grades [108]. More in detail, the authors found that the expression levels of SFRP3 protein were decreased in the nucleus and increased in the cytoplasm of higher grade astrocytoma cells. Such a behavior seemed to be coupled to tumor aggressiveness so that the authors conclude that this protein may have a dual role, being able to act as antagonist or agonist of Wnt signaling [108].

While factors able to inhibit Wnt signals are in general depleted/reduced in GBMs, many other factors are overexpressed and can aberrantly stimulate this pathway in these tumors. One of them is Evi/Wntless/Sprinter/GPR177, a highly conserved seven-domain transmembrane protein that acts as a secretory protein required for exocytosis of Wnt proteins reviewed in [109,110]. Augustin et al. [111] demonstrated that Evi is overexpressed in human gliomas in comparison to normal adult brain tissue and is crucial for glioma cell growth ex vivo and in vivo. In contrast, loss of Evi resulted in downregulation of cell cycle and survival genes. Another important pro-tumoral factor is the WNT target gene FoxM1. The related protein directly binds to β-catenin and promotes its translocation into the nucleus [112]. A very comprehensive review pointed out that the expression level of FoxM1 protein in human glioma tissue is directly correlated with tumor grade and inversely correlated with patient survival. Accordingly, an increased FoxM1 expression in glioma cells enhanced their tumorigenicity, invasiveness, and angiogenesis [89]. Another factor stimulating the activity of the canonical Wnt pathway is pleiomorphic adenoma gene like 2 (PLAGL2), a putative C2H2 zinc finger transcription factor, identified through its structural homology to PLAG1, another member of the same family and a proto-oncogene frequently rearranged and overexpressed in pleiomorphic salivary gland adenomas and lipoblastomas. Noteworthy, PLAGL2 overexpression favors glioma formation and progression [90].

Like PLAGL2, other Wnt modulators appear to be not directly linked to this pathway. For example, SIX3 is a human homolog of the highly conserved sine oculis gene family, an essential transcription regulatory factor involved in eye and fetal forebrain development. Results obtained in a very recent study demonstrated that SIX3 was down-regulated in human glioma tissues and human glioma cells (SHG-44, U251, SF126 and U373-MG) in comparison to normal tissues. Such a down-regulation was coupled to the methylation of its promoter. Further data from the same study also indicated that SIX3 down-regulation contributes to glioma invasiveness, this process being strictly related to the activation of Wnt/β-catenin pathway [113]. Another protein, named PEG3/Pw, involved in the modulation of embryonic development through its activity favoring degradation of β-catenin [114], is suppressed in GSCs [115]. Likewise, the expression levels of a homeobox transcription factor (MSX1) were significantly reduced in GBM in comparison to normal brain tissues. Such a decrease induced mesenchymal transition as characterized by down-regulation of E-cadherin, up-regulation of N-cadherin, and enhanced cell migration. Conversely, MSX1 overexpression inhibited mesenchymal transition and cell migration, hindering the activation of Wnt/β-catenin signaling cascade [116]. In addition, the overexpression of the Spermatogenesis- and oogenesis-specific basic helix-loop-helix transcription factor1 (Sohlh1), a germ cell specific transcription factor that is essential for spermatogonial differentiation and early folliculogenesis, inhibited the cellular proliferation, migration, and invasion in GBM cell lines, whereas Sohlh1 silencing induced an aggressive cellular behavior [117].

Further factors that epigenetically interfere with cancer onset/progression are noncoding RNAs, which can be distinguished in microRNAs (miRNAs) and long noncoding RNAs (lncRNAs). Niyazi.et al., [118], applying a specific selection approach on miRNA expression by microarray data, identified a distinct signature comprising 4 miRNAs (hsa-let-7a-5p, hsa-let-7b-5p, hsa-miR-125a-5p and hsa-miR-615-5p) that was confirmed by quantitative real-time PCR (qRT-PCR) and was present in an age- and sex-matched cohort of GBM patients, obtained from The cancer genome atlas (TCGA), who received standard-of-care treatment. A multivariate analysis revealed that this signature was independent of any prognostic parameters available for that data set. Pathway analysis suggested tumorigenesis-associated processes including Wnt signaling. Further reports demonstrated a relationship between noncoding RNAs and Wnt pathway in GBM growth and invasiveness. For example, the *MIR155* host gene (MIR155HG) and its derivatives miR-155 are one of the best characterized complexes playing a critical role in various pathological processes. Recent data indicate that increased MIR155HG is associated with glioma grade, mesenchymal transition, and poor prognosis by the generation of its derivatives miR-155-5p and miR-155-3p, which interfere with the activity of protocadherins 9 and 7, respectively, two molecules able to inhibit the Wnt/β-catenin pathway, thereby restraining GBM growth [119]. In another study, Li et al. [120] pointed out a high expression of miR-106a-5p and an inverse correlation of these levels and APC mRNAs in GBM tissue. Consequently, inhibition of miR-106a-5p expression weakened the invasive ability of this tumor. In addition, miRNA-603 and miRNA-328 were upregulated in glioma cells; this overexpression activated Wnt/β-catenin signaling [121], even though miR-328 activity was mediated via inhibition of SFRP1 [122]. In contrast, it has been found that miR-577 inhibits GBM growth by decreasing the expression of LRP6 and β-catenin [123]. Moreover, the expression of miR-34a resulted decreased in a graded manner in glioma and derived stem cell lines as compared to normal tissue. The ectopic expression of this miRNA in stem cell lines HNGC-2 and NSG-K16 reduced the proliferative and migratory potential of those cells. This effect was achieved by decreased and increased levels of phosphorylated alpha serine/threonine-protein kinase (AKT) (at ser473) and GSK3β, respectively, leading to enhancement of β-catenin degradation [124].

Therefore, if Wnt inhibition by potential drugs may be considered as a possible new therapeutic tool for gliomas, the relevance of direct members of the Wnt pathway or its up-/down-stream mediators should be carefully evaluated. Moreover, the multiple interactions between Wnt pathway and other compounds acting via tyrosine kinase receptors (like epidermal growth factor or hepatocyte growth factor) cannot be underestimated [100].

## 5. Factors in the Wnt Pathway Considered Potential Targets for Novel Experimental Approach in the Glioblastoma Multiforme Therapy

As previously reported, GBM is characterized by high resistance to the current therapeutic measures represented by combination of radiotherapy and TMZ. Therefore, the patients’ prognosis is poor and the mean survival period is around 1.52-years [125]. Importantly, several phase III clinical trials, wherein other pharmacological agents such as bevacizumab (a recombinant humanized monoclonal antibody directed against the vascular endothelial growth factor (VEGF), a pro-angiogenic cytokine) or cilengitide (a cyclic peptide selective inhibitor of integrins that are important in angiogenesis), failed to show an improvement in overall survival [126,127]. Therefore, there is an urgent need to discover new pharmacological targets to restrain the growth/invasiveness of this tumor.

Some reviews have already pointed out emerging targets for GBM therapy [128,129,130,131]. Here, we investigated the main studies focusing on Wnt pathways, which has been positively correlated to the genesis and progression of GBMs, as confirmed by the altered expression of different members of Wnt associated with a bad prognosis in GBM patients. Therefore, this pathway may represent a novel druggable target (see Table 1).

The list comprises drugs and also some human MSCs, the anti-tumor activity of which is discussed in the text of this review. However, for a wider knowledge of further drugs and natural compounds that have been identified as inhibitors and/or modulators of Wnt/β-catenin signaling pathway, the reader may consult the following reviews [20,100,128,140,141,142].

During the last decade, several reviews have already attempted to classify the existing drugs [20,100,140,141,142], some of which are also reported here. One of these agents is LGK974, a potent inhibitor of the Wnt-specific acyltransferase porcupine (PORCN). This enzyme causes palmitoylation and extracellular secretion of WNT ligands, whereas LGK974 prevents its activity, by disrupting the ligand-driven activation of Wnt pathway. Kahlert et al., recently showed that LGK974 significantly reduced GBM cell line proliferation, clonogenicity, expression of the stem cell marker CD133, while inducing tumor cell differentiation towards a glial phenotype [143]. Interestingly, an open-label Phase 1 clinical trial for various tumor types (head and neck, breast and pancreas cancers, not including GBM), with genetic alterations in the Wnt pathway (ClinicalTrials.gov Identifier: NCT01351103) is currently investigating the clinical effects of this compound [144].

Further pharmacological targets that have been identified as modulators of Wnt pathways are represented by the tankyrase (TNKS) enzymes, which are considered positive regulators of Wnt signal able to promote Axin degradation. Recent data indicate that TNKS inhibitors (TNKSi), at least in colorectal cancer cells, can restore functional signal-limiting destruction complexes [145]. One of these inhibitors, XAV939, can prevent Wnt signal in a GBM cell line (U373) resistant to radiotherapy [146]. Another small molecule, SEN461, decreased viability of cultured glioma cell lines and reduced the volume of subcutaneous implanted xenograft tumor acting as WNT/β-catenin pathway inhibitor, through Axin stabilization and a mechanism partly dependent on TNKS [147]. However, even though these drugs showed to be promising anticancer agents in GBM and related cells at preclinical level, clinical data are not yet available.

Apart from the chemical compounds discussed so far, it is noteworthy that some natural compounds may act as inhibitors and/or modulators of Wnt/β-catenin signaling pathway. A recent report has shown that pyrvinium pamoate (PP), a well-known anthelmintic drug that exhibits a potent antitumor activity against several cancers including GBM [134], acts as a selective WNT pathway inhibitor. Moreover, trichosanthin (TCS), a bioactive protein extracted and purified from the tuberous root of *Trichosanthes kirilowii*, a well-known traditional Chinese medicinal plant, produces antitumor effects in various types of cancer cells, also in those deriving from gliomas. In particular, TCS inhibited the proliferation of glioma cells in a dose- and time-dependent manner and their invasion and migration ability. Further data revealed that the expression levels of LGR5 and of key proteins in the Wnt/β-catenin signaling pathway were markedly decreased after TCS treatment, suggesting that TCS may induce apoptosis in glioma cells by targeting LGR5 and repressing the Wnt/β-catenin signaling pathway [135]. In this context, it has to be underlined that another natural compound, resveratrol, a polyphenolic phytoalexin found in fruits and vegetables, has been shown to inhibit human GSC proliferation and motility, by modulating some factors in the Wnt pathway [83].

A great interest has recently been raised by the phosphatidylinositol-3-kinase (PI3K)/AKT signaling pathway that results to be dysregulated in GBM [132]. Upon activation, AKT can effectively suppress the role of GSK3β, thus leading to a decrease in the degradation of β-catenin that, in turn, elicits a decreased transcription of its target genes resulting in proliferation, inhibition and apoptosis. On this biological basis, Furuta et al. [133] have recently reported that the administration of a cocktail of GSK3β-inhibitory drugs (cimetidine, lithium, olanzapine, valproate, from which the acronym CLOVA), together with TMZ, in a mouse model and in patients with recurrent GBM, significantly inhibited cell invasion and proliferation. Moreover, the patients treated with CLOVA cocktail plus TMZ showed an increased survival in comparison to the control group treated with TMZ alone. Interestingly, the efficacy of this drug in GBM treatment is also improved when combined with sulforaphane (SFN), a member of the isothiocyanate family normally present in consumed cruciferous vegetables. Recent data showed that SFN strengthened TMZ-mediated apoptosis by inhibiting miR-21 via Wnt/b-catenin signaling in GBM cells [136].

Among old drugs that attracted a certain interest as potential anti-cancer agents, there are non-steroidal anti-inflammatory drugs (NSAIDs), which are currently used for treating inflammation, pain and fever. It is widely known that these drugs act through the inhibition of the activity of the cyclooxygenases, enzyme able to form prostaglandin precursors from arachidonic acid. A recent review has comprehensively reported literature about the protective effects of long-term administration of some drugs belonging to this family against colon cancer and potentially other tumor types by interfering both with the COX and the Wnt pathway [137]. A direct link between the 5-lipoxygenase and Wnt signaling pathways has also been found, and is resulted to be essential for the maintenance of leukemic stem cells. Accordingly, genetic and pharmacological inhibition of 5-lipoxygenase, the key enzyme leading to the formation of leukotrienes from arachidonic acid, led to an impairment of Wnt-dependent acute and chronic myeloid leukemic stem cells. Moreover, 5-lipoxygenase in involved in other Wnt-dependent diseases, such as breast cancer, human head and neck squamous cell carcinomas [137]. Based on this evidence, NSAIDs and molecules under development to control the activity of enzymes related to prostaglandins and leukotrienes could be included in the therapeutic strategy to manage GBM overgrowth.

Further drugs that are currently under investigation as potential antitumor agents are PPAR-γ agonists. It is known that in adipocytes, PPAR-γ amplifies differentiation signals and inhibits proliferation by affecting the Wnt/GSK3β/β-catenin pathway [148]. Similar mechanisms involving the Wnt/β-catenin cascade may also occur in GSCs, wherein PPAR-γ activation exerts a growth inhibitory effect and influences self-renewal and stemness programs [138].

Finally, the possible influence exerted by mesenchymal stem cells (MSCs) on Wnt system and GBM growth must be considered, since their application in oncology seems to be in rapid development. Mesenchymal stem cells, initially isolated from bone marrow, are present in a wide range of adult tissues and biological fluids [149,150,151] and possess, besides the ability of self-renewal, multiple differentiation potential and low immunogenicity, a tropism to tumor microenvironment [152,153,154]. For this reason, they are increasingly used for cancer treatment, including GBM [155,156,157]. However, data obtained so far are not univocal. Indeed, some studies indicated that MSCs can promote the progression of tumors such as breast cancer and colon cancer [158,159], whereas other reports showed opposite MSC effect on hepatoma, Kaposi’s sarcoma and breast cancer [160,161,162,163,164]. In addition, the conditioned medium (CM) derived from MSC cultures contains factors able either to prevent carcinogenesis, by inhibiting proliferation [165], or to promote cell tumor proliferation and migration [166]. Interestingly, the MSC-induced pro-tumorigenic effect seems to be regulated by the Wnt/β-catenin signaling in breast cancer [81,164], whereas the inhibition of tumor proliferation occurs by MSC induced secretion of DKK-1, an inhibitor of the same pathway [163,165]. Furthermore, the MSC-derived CM exerts its effect by targeting the Wnt/β-catenin signaling pathway [166]. The influence of MSCs on tumor growth/invasiveness has been examined also in relation to GBM. Therefore, umbilical cord blood-derived MSCs (UC-MSCs) inhibit GBM proliferation, whereas adipose tissue-derived MSCs promote it [167]. The inhibitory effect exerted by UC-MSCs on GSC growth has recently been confirmed and it is likely due to a direct cell-to-cell interaction [139]. However, the CM derived from the same UC-MSCs promotes GSC growth, invasion and migration by a paracrine effect mediated by the CXC chemokine 2. Thus far, the direct or indirect involvement of the Wnt system in the effects mentioned above in relation to GBM has not yet been thoroughly investigated.

## 6. Conclusions

By collecting data reported in the previous sections, the difficulty in managing the WNT system by a pharmacological point of view, considering all the possible factors able to reduce or to increase the activity of this complex pathway in GBM, is evident.

The problems to afford/overcome are multiple and not only strictly related to the mechanism of action of the new potential drugs. Indeed, the blockade of the Wnt system, which in normal tissues control vital cell functions, could produce serious adverse effects in patients, thus limiting their clinical applications. Consequently, it would be helpful to find particular pharmaceutical strategies to make active Wnt antagonists only or prevailingly at the level of tumor cells. Another problem related to the previous one is finding a proper way to administer drugs in GBM, as this tumor is “protected” by the blood–brain barrier (BBB), even though radiation, usually employed in the GBM treatment, disrupts the BBB, facilitating the delivery of therapeutic agents and their achievement of the brain parenchyma [142]. In this regard, even though there is no certain evidence for an involvement of the Wnt system in human glioma angiogenesis, it is known that this pathway regulates expression of VEGF, a key pro-angiogenesis factor in many types of cancer, including malignant glioma [168]. Therefore, future in vitro or in vivo studies should consider the influence of Wnt inhibition on VEGF in glioma cells or on tumor microvascular angiogenesis, respectively, since these events may alter the BBB permeability and, consequently, the passage of drugs up to the tumor.

High lipophilic drugs or pharmaceutical modifications of molecules or new strategy in drug delivery system may improve the efficacy of agents able to inhibit Wnt system. Different approaches have been adopted and they have recently been reviewed by Bianco et al. [169]. One of the most recent strategies is the employment of nanoparticles which are molecules with different chemical characteristics [170] sharing the ability to transport drugs across the BBB. Anti-cancer agents encapsulated into nanoparticles with a size ranging from 10 to 200 nm can be administered either locally or intravenously and show increased solubility, prolonged retention time and stability. By the use of nanoparticles it is also possible to have a more controlled drug release with reduced side effects [171]. Another strategy that is currently under investigation is the use of hydrogels, which are three-dimensional, cross-linked networks of water soluble polymers. They seem to be ideal candidates for local delivery of anti-cancer agents [172]. Two main classes of hydrogel are currently being explored for the treatment of GBM: poly(lactic-*co*-glycolic acid)-based hydrogels [173], and photopolymerizable hydrogels [174].

Additionally, it would be of crucial importance to select patients who can undergo therapy with drugs targeting the Wnt system. As herein reported, gliomas are different for their malignancy and for the correlation of their invasion/aggressiveness with Wnt dysregulation. Therefore, once each tumor has been resected (when possible), it would be mandatory that cultures of GSCs deriving from surgical samples be set up to evaluate the consistence of targeting Wnt pathway to identify which and how many factors in this cascade are dysregulated in those cells. This also means that a bio-bank of tumor samples and GSCs should be constituted, making available tissues and cells to researchers who are investigating on possible new strategies ranging from the identification of markers to pharmaceutical vehicles to deliver drugs at the tumor site. Surely, it would be greatly helpful if these studies could lead to a list of molecules recognized as specific markers for Wnt pathway dysregulation like LEF1 [93] or HOXA13 [94] in GBM. In the future, this would avoid waste of time in setting up cell cultures, thus allowing a direct labeling of tumor cells with Wnt alterations during surgery or peri-surgery, by using selective antibodies against those recognized Wnt linked-tumor markers.

Only by the cooperation among scientists and the exchange of scientific information will the achievement of successful results in the therapeutic management of GBM be possible.

## Figures and Tables

**Table 1:** List of some old and new drugs able to target Wnt pathway that have been shown to be beneficial in experimental glioblastoma multiforme (GBM) therapeutic management, thus representing potential candidates for clinical application.

Target	Compound/Agent	Potential Indication	Source or Previous Indication	Reference
Porcupine	LGK974	GBM cell lines	Chemical compound	[82]
Axin degradation	Tankyrase inhibitors (XAV939, SEN461)	GBM cell line(U373)	Chemical compounds	[132,133]
Wnt/β-catenin	Pyrvinium pamoate	CD133 + GBM initiating cells	Anthelminthic drug	[134]
LGR5	Trichosanthin	Glioma cells	Bioactive protein from *Trichosanthes kirilowii*	[135]
Nuclear β-catenin and c-Myc protein levels	Resveratrol	Human GSCs	Polyphemolic phytoalexin from fruits and vegetables	[83]
GSK3β	Cimetidne + lithium + Olanzapine + valproate (CLOVA cocktail)	Mouse model and patients with GBM	Anti-H2 receptor + antidepressants + anticonvulsant drugs	[133]
miR-21	sulforaphane	Human glioma cell lines	Isothiocyanate compound from cruciferous vegetables	[136]
Wnt pathway	NSAIDs	CSCs other than GSCs	Anti-inflammatory drugs	[137]
Wnt pathway	Anti-leukotrienes	CSCs other than GSCs	Anti-asthmatic drugs	[137]
Wnt/GSK3β/βcatenin	PPAR-γ agonists	GSCs	Anti-diabetic drugs	[138]
Wnt pathway (?)	UC-MSCs	GSCs	Human fluid	[139]
